# Gamma knife radiosurgery for metastatic brain tumors with contrast media leakage: Case series

**DOI:** 10.1097/MD.0000000000041189

**Published:** 2025-01-03

**Authors:** Sue-Jee Park, Wan Park, Yeong Jin Kim, Kanghee Ahn, Kyung-Sub Moon, In-Young Kim, Shin Jung, Correia Dircia Canisia Marcelina, Seul-Kee Kim, Kyung-Hwa Lee, Tae-Young Jung

**Affiliations:** a Department of Neurosurgery, Chonnam National University Medical School and Hwasun Hospital, Seoyang-ro, Republic of Korea; b Department of Radiology, Chonnam National University Medical School and Hwasun Hospital, Seoyang-ro, Republic of Korea; c Department of Pathology, Chonnam National University Medical School and Hwasun Hospital, Seoyang-ro, Republic of Korea.

**Keywords:** brain edema, case report, contrast media, metastasis, radiosurgery

## Abstract

**Rationale:**

The phenomenon of “contrast media leakage” in metastatic brain tumors, where contrast enhancement of perilesional edema can overestimate actual tumor volume.

**Patient concerns and diagnosis:**

The radiologic and pathologic characteristics of 3 surgically resected metastatic brain tumors with contrast media leakage were analyzed. Five metastatic tumors were treated with gamma knife radiosurgery (GKRS), deliberately avoiding areas of contrast media leakage.

**Interventions:**

The characteristics of these tumors, the administered radiation dosage, and progression-free survival were evaluated.

**Outcomes:**

The region of “contrast media leakage within edema” showed different signals from tumor boundaries on T2-weighted magnetic resonance imaging, fluid-attenuated inversion recovery, and apparent diffusion coefficient maps. No increased cerebral blood volume and a low transfer coefficient were indicated on perfusion images. Pathologically, these areas showed prominent endothelial proliferation and perivascular lymphocyte infiltration without tumor cell infiltration. Immunohistochemical staining revealed a weak positive for clauidin-5 and a strong positive with antibodies against leukocyte common antigen and cluster of differentiation 68. Five lesions treated with GKRS were adenocarcinomas of lung origin. The median radiation volume was 3.10 cc (range, 2.32–3.78), and the median radiation dose was 22 Gy (range, 20–22). Treatment responses were nearly complete in 1, partial in 3, and stable in 1. There were recurrences at 6.0 and 10.0 months after GKRS. Median progression-free survival was 18.2 months (95% confidence interval: 9.2–27.1), and there was no treatment-related complication.

**Lessons:**

This study revealed that the region of “contrast media leakage within edema” showed more pronounced blood–brain barrier disruption associated with inflammatory cells. It was effective when the GKRS targeted the actual tumor, excluding the area with contrast media leakage.

## 1. Introduction

The central nervous system contains a specialized dynamic structure known as the blood–brain barrier (BBB), which plays a crucial role in protecting the brain from harmful substances present in the blood and maintaining a homeostatic microenvironment.^[1,2]^ Because of the presence of the BBB, normal brain tissue is not enhanced after contrast media administration. However, pathological contrast enhancement can be observed in pathological conditions in which BBB permeability increases or the BBB is disrupted, such as infarction, inflammation, infection, demyelinating disease, vascular malformations, or tumors.^[3]^ Recently, studies have been conducted on the clinical implications of using brain magnetic resonance imaging (MRI) quantitative analysis to detect subtle BBB dysfunction through contrast leakage, as a tool to aid clinical decision-making.^[4]^

Among these, this study focuses on the phenomenon of “contrast media leakage” in metastatic brain tumors, where contrast leakage into the edematous areas surrounding tumors, without actual tumor presence, potentially resulting in an overestimation of the actual tumor size. The overestimated tumor volume presents significant challenges in treatment planning for gamma knife radiosurgery (GKRS). Increased tumor volume necessitates a reduction in radiation dose, potentially compromising effective tumor control. Furthermore, a larger tumor volume is associated with a higher incidence of complications, including radiation necrosis.

This study aims to differentiate between the tumor and the contrast media leakage through detailed radiologic analysis and examine the pathological findings of areas surrounding the metastatic brain tumors where this leakage occurs. Based on these findings, we aim to assess its implications for GKRS, potentially leading to more accurate treatment planning and improved patient outcomes.

## 2. Material and methods

### 2.1. The radiologic and pathologic analysis of “contrast media leakage”

This study was conducted after obtaining approval from our Institutional Review Board, and written informed consent was obtained from all patients.

Surgical resection was performed on 3 patients exhibiting brain metastases with evidence of contrast media leakage, and their radiological and pathological characteristics were analyzed. Patients preoperatively underwent brain MRI using a Siemens MAGNETOM Vida 3.0 T scanner (MAGNETOM Vida, Siemens, Erlangen, Germany) with a series of sequences including contrast-enhanced T1, T2-weighted, fluid-attenuated inversion recovery (FLAIR), apparent diffusion coefficient (ADC) maps, dynamic susceptibility contrast (DSC), and dynamic contrast-enhanced (DCE) sequences. A standard single dose (0.2 mL/kg) of meglumine gadoterate (Dotarem, 0.5 mmol/mL, Guerbet, 95943 Roissy CdG Cedex, France) was administered intravenously. These techniques facilitated the identification of contrast media leakage within edema, allowing differentiation from the tumor core and adjacent brain tissue. To further characterize these regions, DCE and DSC imaging were performed to obtain quantitative analyses, including cerebral blood volume (CBV) and the volume transfer coefficient (K trans).

**Figure 1.**
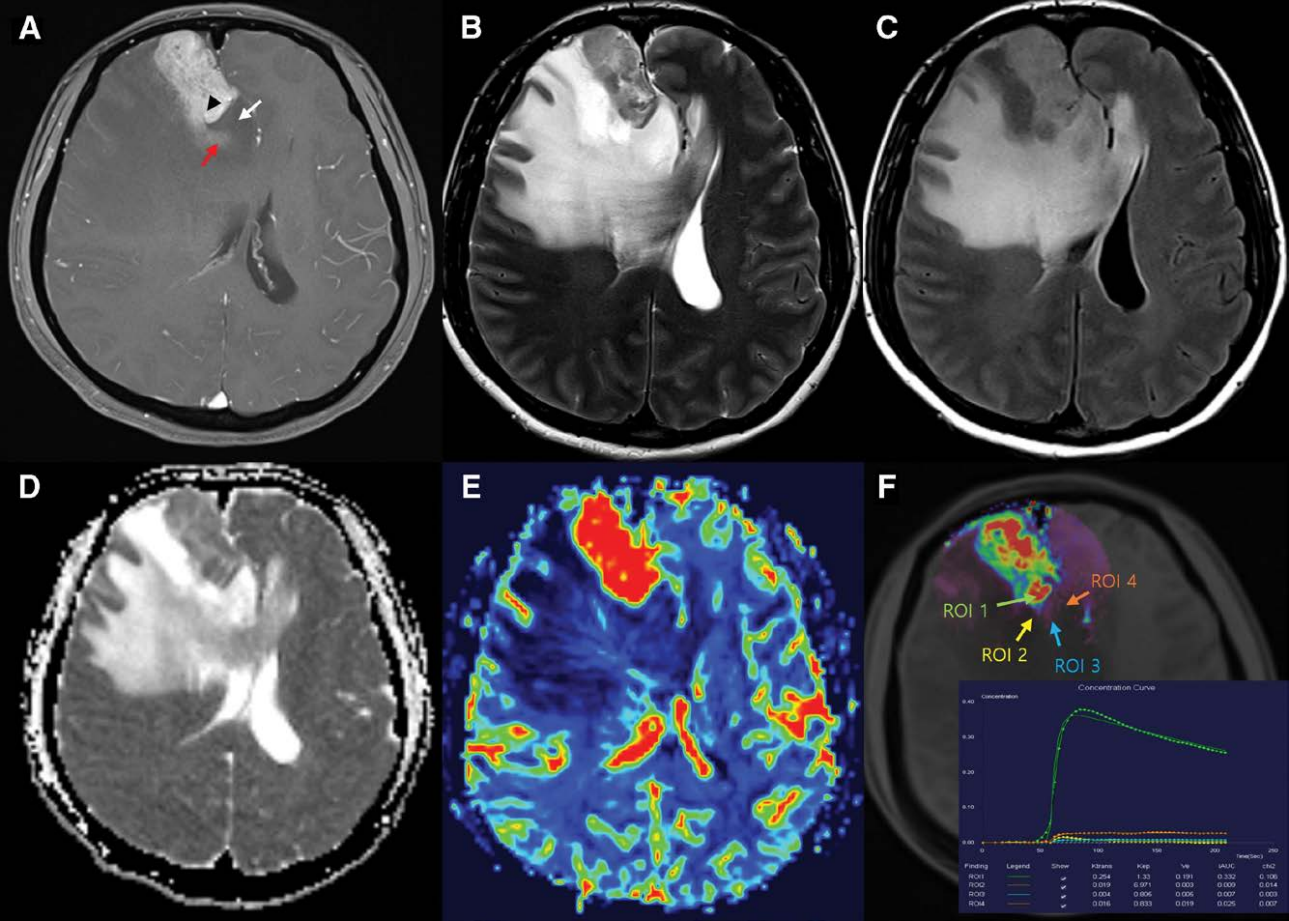
Case 1: radiologic findings of a metastatic brain tumor with contrast media leakage. (A) Contrast-enhanced brain magnetic resonance imaging (MRI) reveals a 4-cm heterogeneously-enhancing mass with contrast media leakage on the right frontal lobe (tumor: arrowhead, contrast media leakage within edema: red arrow, and non-contrast leakage within edema: white arrow). The tumor has iso-to-high intensity signals, and contrast leakage has high-intensity signals similar to edema on (B) T2-weighted MRI and (C) fluid-attenuated inversion recovery (FLAIR). Contrast leakage showed higher values and was distinct from the tumor margin on (D) apparent diffusion coefficient (ADC) maps. (E) Dynamic susceptibility contrast (DSC) and (F) dynamic contrast-enhanced (DCE) reveal the tumor has increased cerebral blood volume (CBV) and a high K trans value and contrast leakage with a low CBV and low K trans value [tumor: region of interest (ROI) 1, contrast media leakage within edema: ROI 2, 3, non-contrast media leakage within edema: ROI 4].

All patients underwent a neuronavigation-assisted surgical craniotomy and tumor resection under general anesthesia. The neuronavigation was guided by contrast-enhanced T1-weighted images, which were co-registered with T2-weighted, FLAIR, or ADC sequences for accurate localization. Biopsies were taken from 3 areas: the tumor itself, the region of contrast media leakage within the edema, and the region of non-contrast leakage within the edema (as indicated in Fig. 1A).

Three samples in the “tumor itself,” 5 in the region of “contrast media leakage within edema,” and 13 in the region of “non-contrast leakage within edema” from 3 patients with brain metastases were examined histologically. Selected histological sections were stained with hematoxylin–eosin and immunostained using anti-cytokeratin, anti-claudin-5 (CLDN 5), anti-leukocyte common antigen, and anti-cluster of differentiation 68 antibodies. The histological interpretation was performed by a specialized neuropathologist.

### 2.2. Gamma knife radiosurgery for 5 metastatic brain tumors with contrast media leakage

Five cases of metastatic brain tumors were treated with GKRS using Leksell GammaPlan^®^ radiosurgery planning software, deliberately avoiding the distant regions of contrast media leakage. We described various clinical factors, including age, sex, the pathological type of each tumor, tumor volume at the time of GKRS, tumor location, delivered radiation dose, the percentage of the isodose line, and any subsequent systemic treatments. The efficacy of GKRS in managing metastatic brain tumors was assessed by analyzing treatment response, progression-free survival (PFS), and overall survival (OS). PFS was measured from the date of GKRS until the occurrence of recurrence or last follow-up. OS was calculated from the date of GKRS to the date of death or last follow-up. Furthermore, complications, including radiation toxicity, were also evaluated.

## 3. Results

### 3.1. The radiologic and pathologic characteristics of contrast media leakage

#### 3.1.1. Case 1

A 46-year-old female patient with a history of breast cancer presented with a brain metastasis. The brain MRI revealed a 4 cm heterogeneously enhancing mass in the right frontal lobe, with contrast media leakage around the tumor observed in contrast-enhanced T1-weighted images (Fig. 1A). The tumor exhibited iso-to-high intensity signal on T2-weighted and FLAIR images (Fig. 1B and C), with the region of “contrast media leakage within the edema” appearing highly intense, similar to the surrounding edema. In ADC sequences, the region of “contrast media leakage within edema” exhibited higher values, distinct from the tumor (Fig. 1D). Additionally, the tumor demonstrated high CBV on DSC imaging and a high K trans value on DCE imaging. Conversely, the region of “contrast media leakage within edema” displayed components with low CBV and K trans values (Fig. 1E and F).

**Figure 2.**
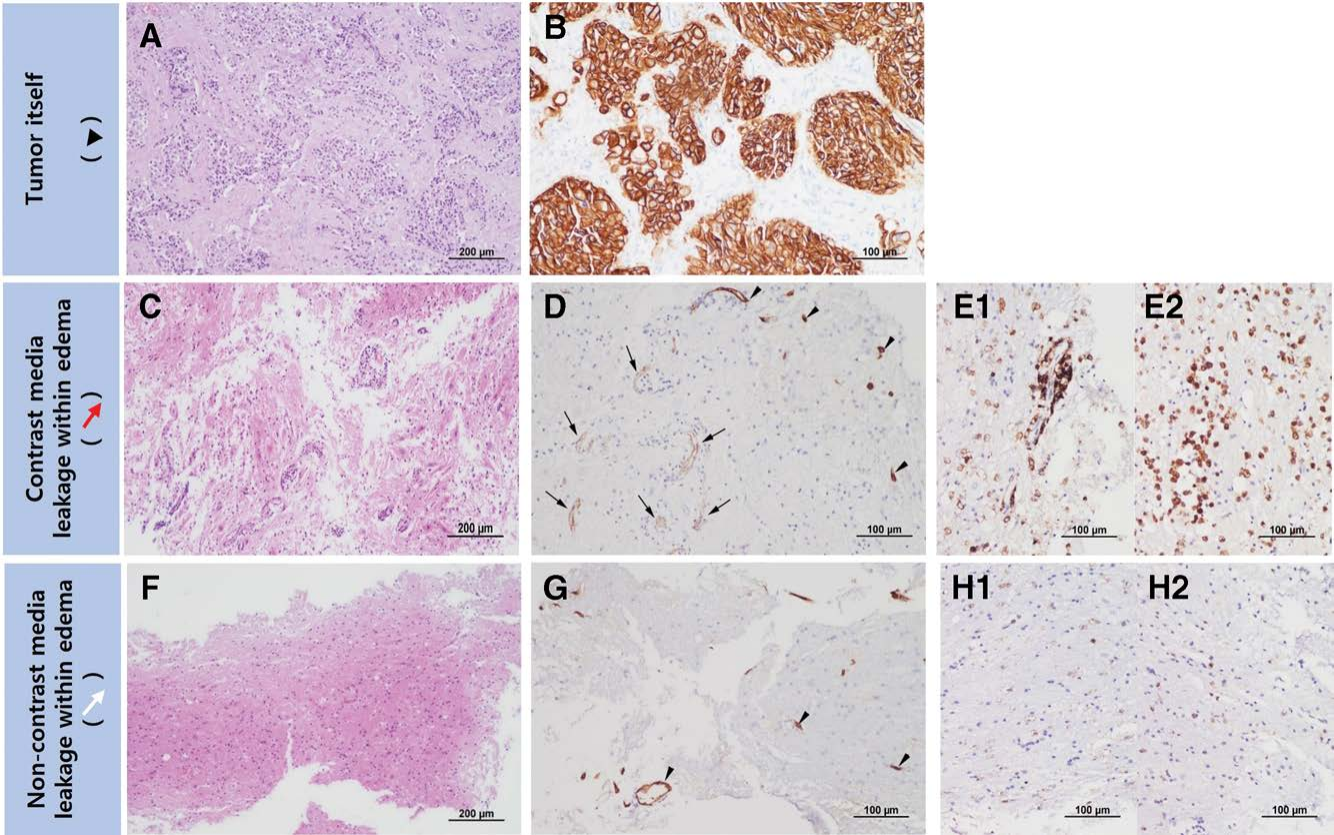
Case 1: pathologic findings of a metastatic brain tumor with contrast media leakage. A and B (Tumor): The tumor is composed of densely populated epithelial components interspersed within the stroma on (A) hematoxylin and eosin (H/E) staining (magnification ×100) and strongly positive for (B) anti-cytokeratin (magnification ×200), a diagnostic marker of metastatic carcinomas. C–E (Region of contrast media leakage within edema): Abnormal endothelial proliferation and perivascular lymphocyte infiltration are shown by (C) H/E staining (magnification ×100). The region shows some vessels with decreased immunopositivity for anti-CLDN5 (arrows) compared to the vessels with well-preserved CLDN5 immunoreactivity (arrowheads) (D). The region is strongly positive for (E1) anti-LCA and (E2) anti-CD68 (magnification ×200) by immunohistochemistry. F–H (Region of non-contrast media leakage within edema): Normal endothelial cells and no perivascular lymphocyte infiltration are present by (F) H/E staining (magnification ×100). The region is strongly immunopositive (arrowheads) for (G) anti-CLDN5 antibody and sparsely immunopositive for (H1) anti-LCA and (H2) anti-CD68 antibody (magnification ×200). CD68 = cluster of differentiation 68, CLDN5 = clauidin-5, H/E = hematoxylin and eosin, LCA = leukocyte common antigen.

Histologically, the tumor showed varied epithelial cells with cytoplasmic vacuoles on H/E staining (Fig. 2A), and strong cytokeratin expression confirmed its metastatic breast origin (Fig. 2B). In the region of “contrast media leakage within edema,” there was abnormal endothelial proliferation and lymphocyte infiltration (Fig. 2C), claudin degradation indicating compromised tight junctions (Fig. 2D) and marked immune cell presence (Fig. 2E). Conversely, the region of “non-contrast media leakage within edema” maintained normal endothelial cells with intact tight junctions (Fig. 2F and G) and sparse immune cell infiltration (Fig. 2H). No tumor cells were found in either the region of contrast media leakage or non-contrast media leakage within the edema.

#### 3.1.2. Cases 2 and 3

Patients with lung and gastric cancer each developed brain metastases in the left temporal and right parieto-occipital lobes, respectively. MRI findings were similar to case 1, showing heterogeneously enhancing masses with contrast media leakage (Fig. S1A and E, Supplemental Digital Content, http://links.lww.com/MD/O260). The regions of ``contrast media leakage within edema’’ showed distinct signal patterns from the tumor on T2-weighted MRI, FLAIR, and ADC maps, resembling the signal patterns of peritumoral edema (Fig. S1B–D and F–H, Supplemental Digital Content, http://links.lww.com/MD/O260). Pathologically, these cases paralleled the findings in case 1, with the region of ``contrast media leakage within edema’’ showing abnormal endothelial proliferation, disrupted tight junctions evidenced by weak clauidin-5 immunopositivity, and significant immune cell infiltration, especially marked by leukocyte common antigen and cluster of differentiation 68 (Fig. S2A–C and G–I, Supplemental Digital Content, http://links.lww.com/MD/O260). Regions of ``non-contrast media leakage within edema’’ appeared relatively normal endothelial cells and intact tight junctions (Fig. S2D–F and J–L, Supplemental Digital Content, http://links.lww.com/MD/O260).

### 3.2. Gamma knife radiosurgery for metastatic brain tumors with contrast media leakage

The characteristics of the 5 tumors are summarized in Table 1. The male-to-female ratio was 4:1, and the median age was 64 (range 57–70). The tumor locations were distributed as follows: 2 patients had tumors in the frontal lobe, 1 in the occipital lobe, 1 in the temporal lobe, and 1 in the parietal lobe. The pathological types of tumors were all adenocarcinomas from lung cancer. The median target volume was 3.1 cc (range, 2.32–3.78 cc). The marginal prescription doses were 20 Gy for 2 patients and 22 Gy for 3 patients. The prescription isodose percentage was 50% in 4 patients and 75% in 1 patient. The number of patients who received cytotoxic systemic chemotherapy for lung cancer after GKRS was 2; 2 patients received immunotherapy, and 1 received targeted molecular therapy. After GKR, the mean PFS of the 5 tumors was 18.2 months (95% confidence interval: 9.2–27.1, range: 6.0–26.9), and the mean OS was 25.1 months (95% confidence interval: 10.9–39.2, range: 6.1–54.4) in 5 patients. No complication, including radiation necrosis, was observed.

**Table 1:** Characteristics and clinical outcomes of 5 patients who underwent GKRS.

GKRS case	Age/sex	Location	Type of pathology (primary ca.)	Tumor volume	Marginal dose (isodose %)	Systemic tx.	Clinical outcomes		
							PFS (months)	OS (months)	Cause of death
1	70 /M	Lt. T	Adenocarcinoma (lung)	3.10	22 Gy (50%)	Keytruda® immunotherapy	26.9 (no recurrence)	26.9 (expire)	Lung cancer
2	68 /M	Rt. F	Adenocarcinoma (lung)	2.32	20 Gy (75%)	Keytruda® immunotherapy	6.0 (recurrence: re-GKRS)	54.4 Follow-up	Lung cancer
3	64 /M	Rt. O	Adenocarcinoma (lung)	3.35	22Gy (50%)	Cytotoxic chemotherapy	6.1 (no recurrence)	6.1 (expire)	Lung cancer
4	59 /M	Rt. P	Adenocarcinoma (lung)	3.78	22Gy (50%)	Cytotoxic chemotherapy	17.8 (no recurrence)	17.8 (expire)	Lung cancer
5	57 /F	Rt. F	Adenocarcinoma (lung)	2.75	20Gy (50%)	Iretinib® targeted therapy	10.0 (recurrence: resection)	20.2 (expire)	Lung cancer

Ca. = cancer, F = female, F = frontal lobe, GKRS = gamma knife radiosurgery, Lt. = left, M = male, O = occipital lobe, OS = overall survival, P = parietal lobe, PFS = progression free survival, Rt. = right, T = temporal lobe, Tx. = treatment.

In case 2, 1 patient had a PFS of 6.0 months after GKRS. This patient was in stable condition after achieving a partial response. However, they underwent repeated GKRS due to a regrowing mass. In case 5, the PFS was 10.0 months. This patient required surgical resection due to tumoral bleeding with recurrence.

### 3.3. A representative GKRS-case1 for brain metastasis with contrast media leakage

**Figure 3.**
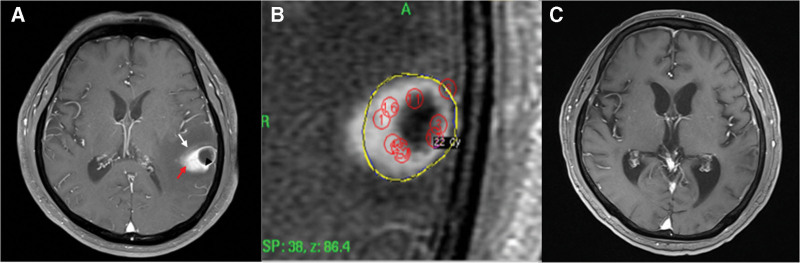
A representative GKRS-case1 for brain metastasis with contrast media leakage. (A) Contrast-enhanced brain MRI reveals a 3.4-cm heterogeneously-enhancing mass with contrast media leakage on the left temporal lobe (tumor: arrowhead, contrast media leakage within edema: red arrow). (B) GKRS was performed on the tumor with a marginal prescription dose of 22 Gy at 50% prescription isodose, deliberately avoiding the distant regions of contrast media leakage. (C) The enhancing mass had achieved complete remission on brain MRI. GKRS = gamma knife radiosurgery, MRI = magnetic resonance imaging.

A 70-year-old male patient had a 3.4 cm heterogeneously enhancing mass with contrast media leakage in the left temporal lobe on contrast-enhanced T1-weighted MRI (Fig. 3A). This was an asymptomatic, metachronous metastatic tumor from adenocarcinoma of the lung. GKRS was administered for a tumor volume of 3.1 cc with a marginal prescription dose of 22 Gy, deliberately avoiding the distant regions of contrast media leakage (Fig. 3B). The prescription isodose was 50%. After GKR, immunotherapy was administered for lung cancer for 2 years. After GKR, immunotherapy was administered for lung cancer for 2 years. Seven months after GKRS, the enhancing mass had decreased and achieved complete remission without any complications, as seen on brain MRI (Fig. 3C). There was no recurrence for 26.9 months.

## 4. Discussion

The tumors produce vascular endothelial growth factor (VEGF) that helps blood supply to maintain their growth and survival. As VEGF is secreted, the tumor becomes neovascularized without the BBB, and the tumor itself secretes cytokines or proteins to damage the tight junction or basement membrane, ultimately leading to BBB breakdown.^[3,5–8]^ Therefore, when contrast-enhanced MRI is performed, the tumor is enhanced as the contrast media penetrates through the capillaries where the BBB is disrupted.^[3]^ In addition, when permeability increases due to BBB breakdown, serum proteins such as albumin and immunoglobulin G also extravasate into the interstitium. When serum proteins enter the interstitium, the interstitial oncotic pressure rises, leading to the accumulation of salt and proteinous fluid in the surrounding tissues, which causes vasogenic edema around the tumor.^[9,10]^

However, despite increased BBB permeability in the edema surrounding the tumor, contrast media leakage did not occur in all peritumoral edema, and it was occasionally seen in edema surrounding some metastatic brain tumors. Therefore, a crucial clinical question is whether “the region of contrast media leakage within edema” is actually enhanced by tumor cell infiltration. This question is crucial when planning the treatment boundary using stereotactic radiosurgery or surgery. This is the reason for determining whether to include the contrast media leakage region in the resection range when it is adjacent to an eloquent area, or whether to designate the leakage region as a target during GKRS, where the radiation dose is planned based on tumor volume. In addition, an overestimated tumor volume may be associated with a risk of radiation necrosis.^[11]^ According to the results of our study, no tumor cell infiltration was detected in the regions of “contrast media leakage within edema” surrounding metastatic brain tumors.

As the region of “contrast media leakage within edema” is not actually infiltrated by the tumor, it was considered distinguishable from tumor tissue characteristics, such as cellularity, water or fat composition, and necrosis. Each imaging sequence, including T2-weighted MRI, FLAIR, ADC, DSC, and DCE, was analyzed accordingly. As shown in our cases, radiologic findings revealed that the signal exhibited by contrast media leakage was notably distinct from that of the tumor on MRI. Especially, the region of “contrast media leakage within edema” showed tumor-like enhancement on contrast-enhanced T1-weighted MRI, but showed an edema-like signal rather than a tumoral signal on T2-weighted MRI, FLAIR, and ADC maps. Similar to the utilization of DSC and DCE techniques in distinguishing between tumor recurrence and radiation necrosis, these imaging modalities can also assist in differentiating contrast media leakage from tumors.^[12]^ DSC images revealed an increased CBV in metastatic tumors, whereas no elevated CBV was observed in the region of “contrast media leakage within edema.” Additionally, DCE images showed a high K trans value in the tumor and a low K trans value in the region of “contrast media leakage within edema.”

Furthermore, our study revealed pathological evidence of abnormal endothelial cell proliferation in the region of “contrast leakage within edema.” Additionally, the degradation of claudin proteins, which constitute the tight junction, and infiltration of immune cells such as lymphocytes and macrophages were observed. Conversely, the region of “non-contrast leakage within edema” contained relatively normal endothelial cells, maintained tight junction integrity, and exhibited lower levels of immune cell infiltration, compared to the region of “contrast leakage within edema.” These findings confirm that the region of “contrast media leakage within edema” indicates a more severe disruption of the BBB compared to the region of “non-contrast media leakage within edema.”

Unlike the tumor itself, the region of “contrast leakage within edema” surrounding the tumor exhibits low K trans and CBV values due to the absence of true tumor infiltration. Pathologically, however, this region shows more severe BBB disruption, characterized by endothelial cell proliferation and compromised tight junctions, compared to other edematous regions. Consequently, some contrast media leakage is observed in this region, consistent with both pathological and radiological findings. Although located within the same peritumoral edema, contrast media leakage due to pronounced BBB disruption occurs only in specific regions.

When vasogenic edema occurs around a tumor, it is associated with the development of interstitial oncotic pressure, leading to the occlusion of small vessels and local hypoperfusion.^[9]^ Hypoperfusion, in turn, triggers the up-regulation of angiogenesis-related factors such as VEGF, matrix metalloproteinase-9, and angiopoietin-2, resulting in abnormal endothelial proliferation.^[13–16]^ Furthermore, as hypoperfusion due to peritumoral edema worsens and progresses to ischemia, it leads to the degradation of tight junction proteins such as occludin and claudin and exacerbates the formation of potentially cytotoxic edema. This cytotoxic edema can induce endothelial cell death, thereby increasing BBB permeability.^[17–20]^ Additionally, ischemia can give rise to focal ischemic cell damage, which triggers an inflammatory response, leading to the infiltration of immune cells into the affected area.^[21]^ These infiltrating immune cells release cytokines and chemokines, promoting the aggregation of immune cells.^[22,23]^ This aggregation establishes a self-perpetuating cycle characterized by ongoing cytokine and chemokine secretion, subsequently increasing BBB permeability.^[24–26]^ Based on these findings, we hypothesized that local hypoperfusion would occur within the edema surrounding the tumor leading to ischemia and subsequent ischemic damage-induced inflammation. These processes would contribute to a more pronounced breakdown of the BBB. With the increased BBB permeability, there is potential for contrast media leakage from the blood into the edematous brain parenchyma surrounding the tumor.

In our study, performing GKRS while excluding the areas of contrast media leakage resulted in a PFS of 18.2 months, which could be considered clinically significant and effective. Therefore, if the target tumor volume, including the contrast leakage area, is large, treatment planning is conducted by excluding the contrast leakage region to minimize unnecessary radiation exposure, thereby reducing complications such as radiation necrosis, and simultaneously ensuring effective tumor control by delivering a sufficient radiation dose. When the tumor volume is small, including the contrast leakage area may not be as significant; however, this approach is particularly applicable when treating larger tumors.

This study is limited by a small and heterogeneous sample size, making it premature to generalize the findings. However, additional cases of contrast media leakage should be analyzed and explored to help establish evidence-based recommendations for patients with metastatic brain tumors exhibiting contrast media leakage.

## 5. Conclusion

Contrast media leakage, which shows enhancement of contrast media within the edema surrounding metastatic brain tumors, demonstrates prominent BBB disruption based on our study. Additionally, there is no actual tumor cell infiltration in the region of “contrast leakage within the edema” surrounding the metastatic tumor. Based on these findings, it was confirmed that performing GKRS while excluding the areas of contrast media leakage effectively controls the tumor. Therefore, planning an optimal clinical treatment boundary is crucial to minimize damage to the normal brain parenchyma and ensure appropriate treatment management.

## Author contributions

**Conceptualization:** Sue-Jee Park, Kyung-Hwa Lee, Tae-Young Jung.

**Data curation:** Sue-Jee Park, Wan Park, Tae-Young Jung.

**Formal analysis:** Sue-Jee Park, Tae-Young Jung.

**Writing – original draft:** Sue-Jee Park, Wan Park, Kanghee Ahn.

**Writing – review & editing:** Yeong Jin Kim, Kyung-Sub Moon, In-Young Kim, Shin Jung, Correia Dircia Canisia Marcelina, Seul-Kee Kim, Kyung-Hwa Lee, Tae-Young Jung.

## Supplementary Material


