# Supplementary material for: Gamma knife radiosurgery for metastatic brain tumors with contrast media leakage: Case series
Source: Medicine (Baltimore). 2025 Jan 3;104(1):e41189. doi: 10.1097/MD.0000000000041189 (PMC11709185; doi:10.1097/MD.0000000000041189)
Supplement: Supplementary file 1 [file medi-104-e41189-s001.docx]

**SUPPLEMENTAL DIGITAL CONTENT**

**Figure S1 Case 2,3: radiologic findings of metastatic brain tumors with contrast media leakage**

**
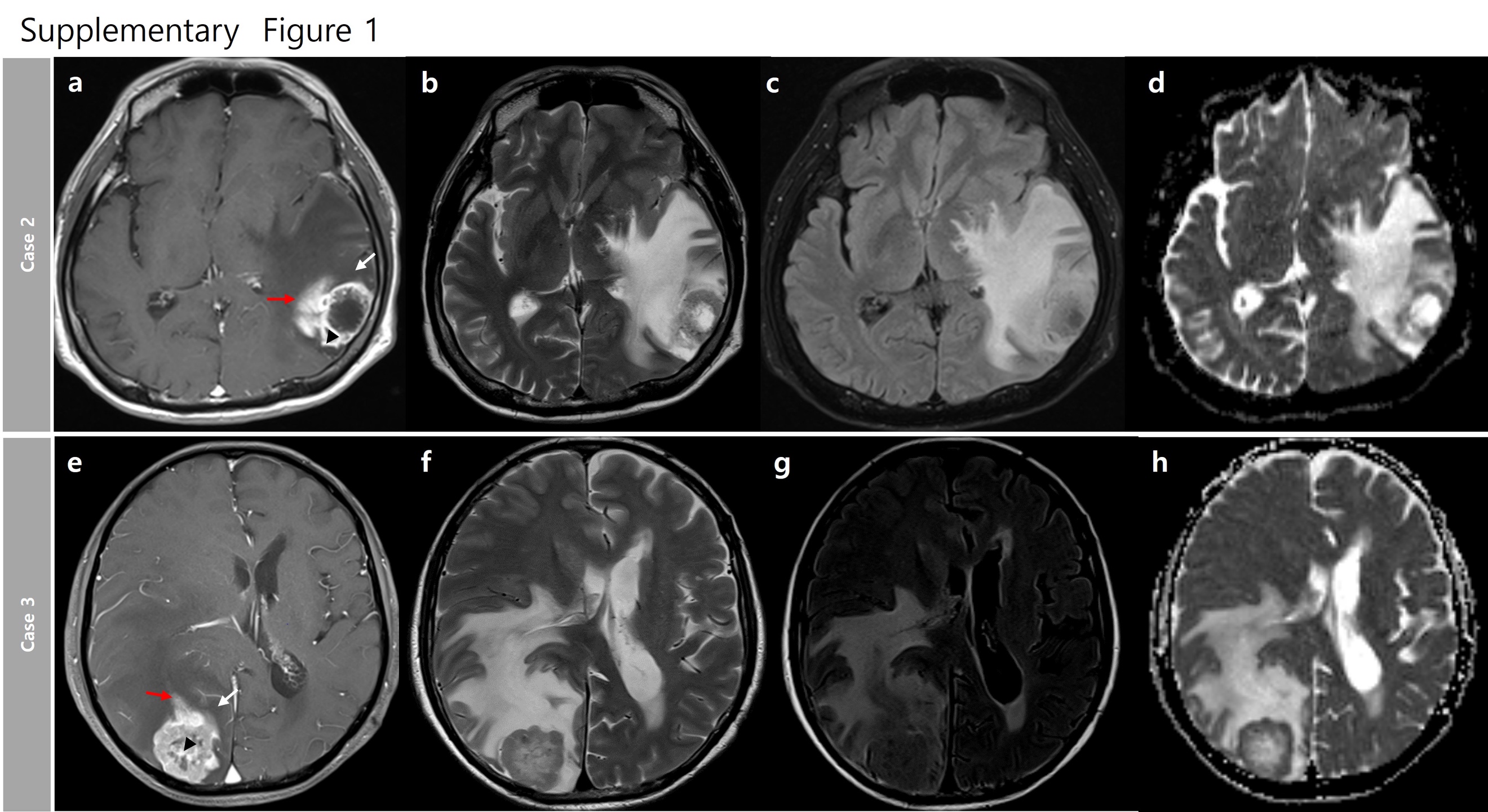
**

**SUPPLEMENTAL DIGITAL CONTENT**

**Figure S2. Case 2,3: pathologic findings of metastatic brain tumors with contrast media leakage**

**
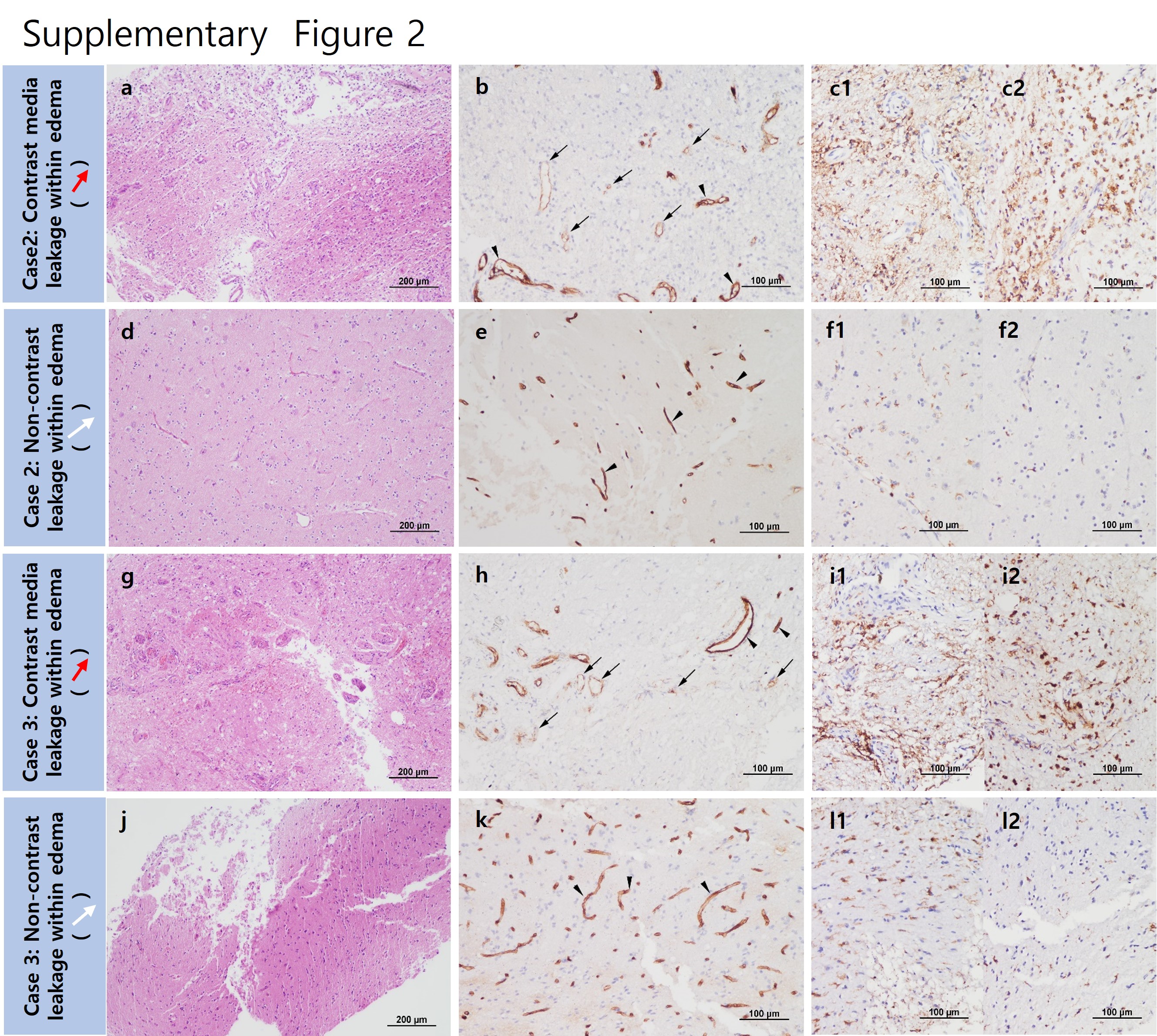
**
